# Classification of Mass Spectral Data to Assist in the Identification of Novel Synthetic Cannabinoids

**DOI:** 10.3390/molecules29194646

**Published:** 2024-09-30

**Authors:** Kristopher C. Evans-Newman, Garion L. Schneider, Nuwan T. Perera

**Affiliations:** Department of Chemistry and Physics, Western Carolina University, Cullowhee, NC 28723, USA

**Keywords:** novel synthetic cannabinoids (NSCs), binary classification system, mass spectral data, partial least square discriminant analysis (PLS-DA)

## Abstract

Detection and characterization of newly synthesized cannabinoids (NSCs) is challenging due to the lack of availability of reference standards and chemical data. In this study, a binary classification system was developed and validated using partial least square discriminant analysis (PLS-DA) by utilizing readily available mass spectral data of known drugs to assist in the identification of previously unknown NCSs. First, a binary classification model was developed to discriminate cannabinoids and cannabinoid-related compounds from other drug classes. Then, a classification model was developed to discriminate classical (THC-related) from synthetic cannabinoids. Additional models were developed based on the most abundant functional groups including core groups such as indole, indazole, azaindole, and naphthoylpyrrole, as well as head and tail groups including 4-fluorobenzyl (FUB) and 5-Fluoropentyl (5-F). The predictive ability of these models was tested via both cross-validation and external validation. The results show that all models developed are highly accurate. Additionally, latent variables (LVs) of each model provide useful mass to charge (*m*/*z*) for discrimination between classes, which further facilitates the identification of different functional groups of previously unknown drug molecules.

## 1. Introduction

Novel psychoactive substances (NPSs) are increasingly reported in recent years and pose significant risks to public health worldwide [1,2]. These substances, sometimes known as “legal highs”, are newly designed drugs that mimic the effects of commonly abused drugs and comprise drug classes including opioids, cathinones, cannabinoids, stimulants, and benzodiazepines. Many NPSs share similar chemical structures with commonly abused drugs and produce alike psychoactive responses by binding to similar receptors in the body. These NPSs are often designed to circumvent the regulations that limit the use of recreational drugs, evade detection during routine analysis, and create more potent drugs [1,2,3]. They became popular worldwide around the late 2000s, and over 1150 NPS compounds have been reported to the United Nations Office on Drugs and Crime (UNODC) Early Warning Advisory (EWA) by 137 countries and territories by October 2022 [4]. According to the Center of Forensic Science Research and Education (CFSRE), there were 21 NPSs reported for the first time in the USA in 2022 to its NPS Discovery Program [5].

In a typical forensic laboratory analysis, an analyst uses a panel of known drug standards or reference materials to identify and quantify drugs present in a sample (or evidence) using chromatographic methods such as gas chromatography–mass spectrometry (GC-MS) or liquid chromatography–mass spectrometry (LC-MS). If a compound present in the sample is not included in the panel, mass spectral libraries can be used to find the identity of that compound by comparing the mass spectrum of the unknown with the mass spectra of known compounds present in the library. These libraries are continuously updated to include NPSs that are identified by various institutions such as crime laboratories [6]. However, new structurally diverse NPSs are introduced each year to the ever-changing illicit NPS market.

In the case of a new NPS, such as a newly synthesized cannabinoid, that has not been reported before, since there are no reference materials or reference spectra available, crime labs rely on intelligence data, prior knowledge of NPSs, and additional analysis methods such as nuclear magnetic resonance spectroscopy (NMR), Fourier transform infrared spectroscopy (FT-IR), or high-resolution mass spectral data (HRMS) to determine the presence of such a compound. However, structural elucidation methods of novel compounds can be expensive and time-consuming. In most crime laboratories, gas chromatography coupled with mass spectrometry that uses electron impact ionization (GC-EI-MS) is the method of choice in seized drug analysis. Some research suggests the possibility of elucidation of structures of these NPSs by studying the possible combinations of molecular fragments that resulted during the ionization of molecules in mass spectroscopic instruments. By evaluating the mass spectra of a class of drugs, one can identify commonly observed ions and relate them to the mass spectral trends that can be used to identify the class of an unknown drug [1]. However, these efforts are highly dependent on the expertise of the analyst and a thorough review of available data. Thus, there is a growing interest in developing methods that can proactively and promptly determine the presence of NPSs in evidentiary material using machine learning methods [7,8,9,10].

A recent review conducted by Feeny and coworkers reported the relationship between the drug class and most abundant ions in their mass spectral data. Although most drug molecules in a drug class contain ions that can be traced to the fragmentation patterns, the presence of a large number of other ions in the majority of class members is not well explained. We postulate that some of these ions may play an important role in discriminating drug classes. Furthermore, the attachment of certain functional groups such as methyl or fluorine, for example, would significantly change the mass-to-charge ratios of commonly occurring peaks such as the base peak. Although this can be predicted, the attachment of a wide variety of functional groups in different positions of the core structure would make prediction difficult. To address this issue other workers reported the development of classification models for fentanyl derivatives [7,9], and other drug classes [10,11]. However, the development of a classification system to address novel synthetic cannabinoids (NSCs) is still lacking.

Synthetic cannabinoids are designed compounds that mimic the effects of naturally occurring cannabinoids, known as endocannabinoids, in the body. These compounds are mixed with plant matter or incense to smoke or a liquid that can be vaporized [5]. Both natural and synthetic cannabinoids have an affinity to the CB1 and CB2 receptors, commonly referred to as cannabinoid receptors, primarily in the brain but found throughout the body as a part of the endocannabinoid system that is responsible for homeostasis and neurotransmitter communication. Thus, these synthetic compounds are sometimes referred to as synthetic cannabinoid receptor agonists (SCRAs). These receptors are a part of a unique system called the endocrine system where our body produces naturally occurring endocannabinoids. They made their street debut in 2004 when JWH-018 was encountered in Europe [12].

**Figure 1:**
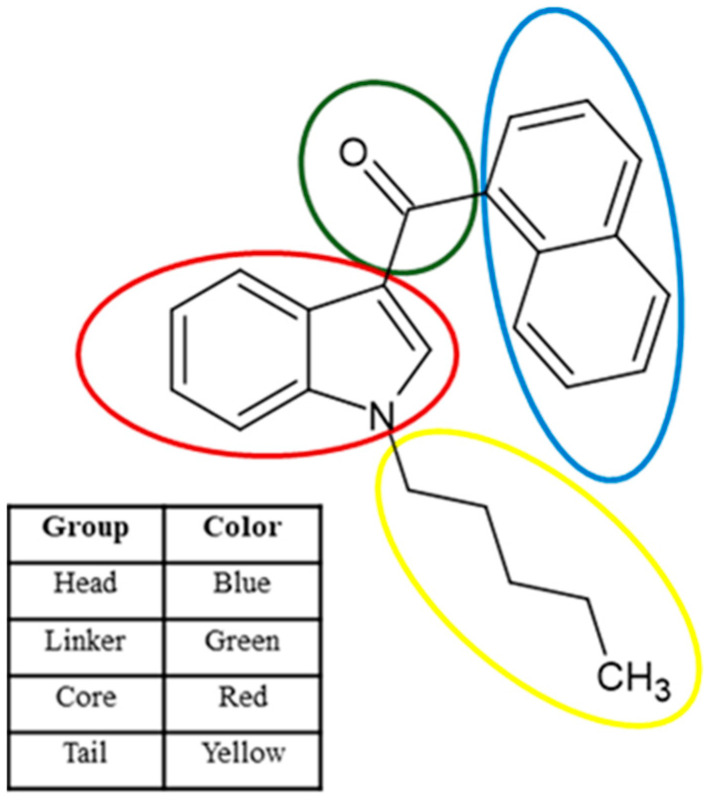
Structure of JWH-018 demonstrating the major groups of a typical synthetic cannabinoid.

Synthetic cannabinoid nomenclature starts either with the initials of the chemist who synthesized them or common names that are abbreviations derived from the International Union of Pure and Applied Chemists (IUPAC) naming system. The IUPAC names of synthetic cannabinoids are impractical due to their length due to the complex structures of synthetic cannabinoids. The abbreviation should capture all key functional groups of the synthetic cannabinoid [13]. Structurally, these compounds consist of four unique groups: head, linker, core, and tail groups (see Figure 1) [12].

Novel synthetic drugs such as NSCs possess a unique problem in classification using machine learning methods. The substitutions are made on multiple positions of existing drug molecules by attaching different structural moieties to make novel drugs. In the case of cannabinoids, the drug designers can make structural changes to the head group, core group, linker group, and/or tail group to make new molecules. When developing classification models, it was found by our research group that the binary classification models work best for a given functional group rather than developing models with multiple classes. We hypothesize that the spectral variance arising from these different structural moieties is large resulting in the models being less accurate. Therefore, binary models were used in this study to determine if a particular functional group is present or absent on an unknown NSC.

**Figure A1:**
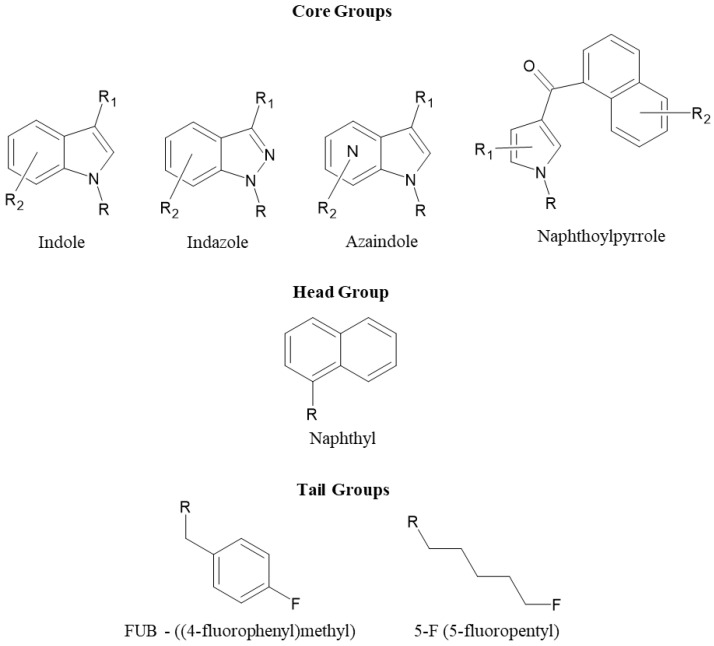
Functional groups of primary interest in the current study.

In this current study, a classification system was developed to identify previously unknown NSCs using PLS-DA. Principal component analysis (PCA) was used to visualize the data space and detect any outliers while hierarchical cluster analysis (HCA) was used to determine the trends of data and identify potential cannabinoid subclasses based on chemical structure. Figure A1 shows common structural groups found in synthetic cannabinoids that are target groups in this study.

## 2. Results and Discussion

### 2.1. Preliminary Evaluation of Data

**Figure 2:**
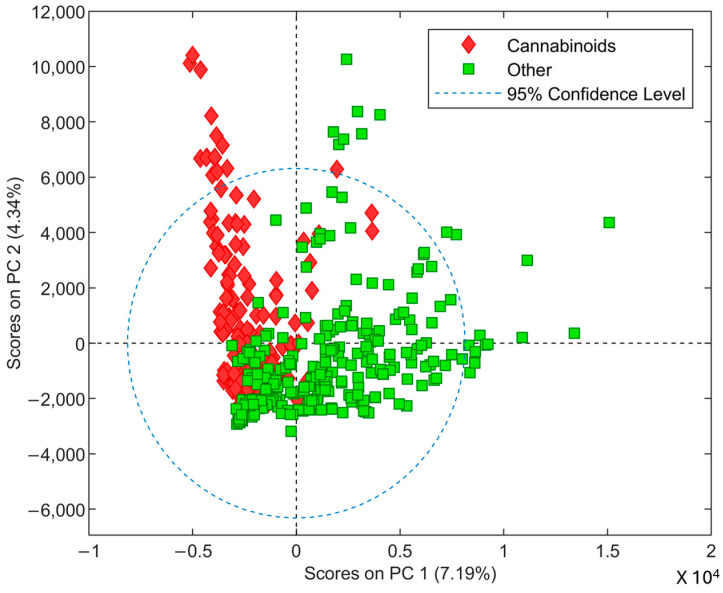
PCA score plot of cannabinoids (Red) and other drug classes (Green).

**Figure A2:**
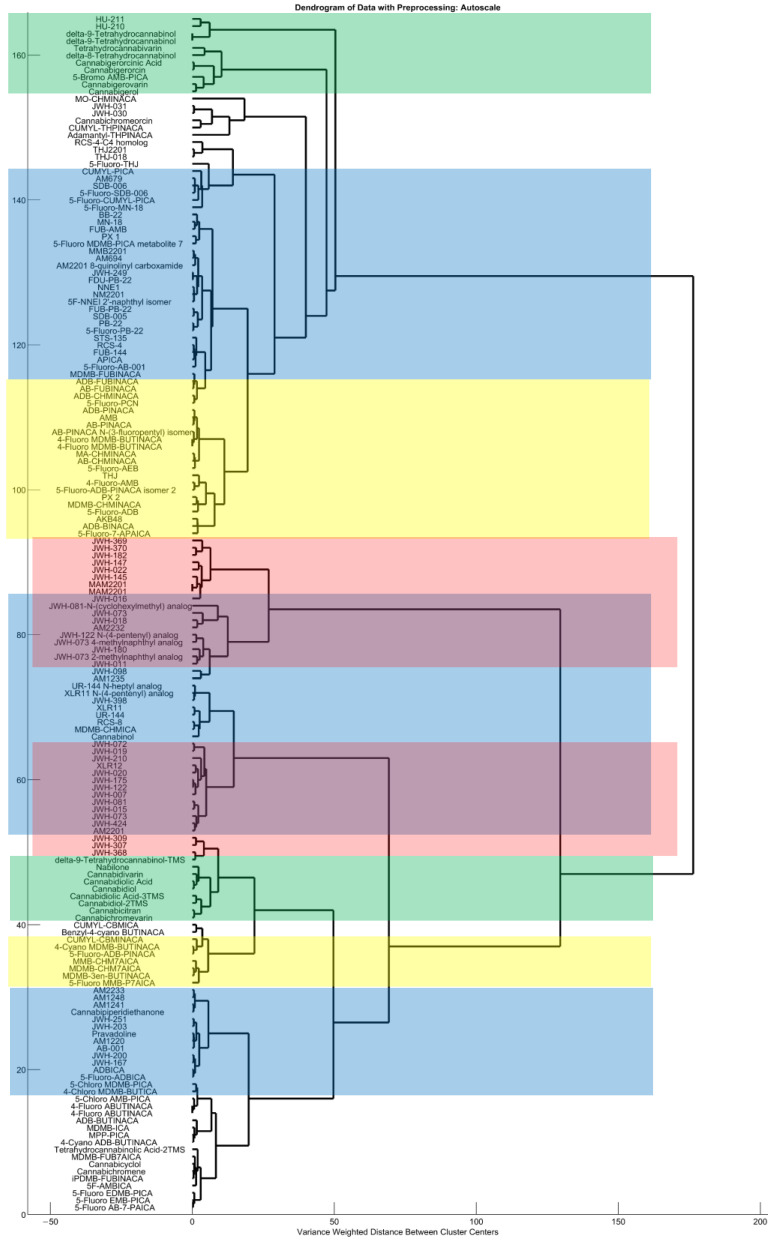
Dendrogram showing the results of HCA. Green: mostly classical cannabinoids, Red: mostly naphthyl head group containing cannabinoids, Blue: mostly indole core containing cannabinoids, and Yellow: mostly azaindole/indazole core containing cannabinoids.

Mass spectral data were obtained from freely available GCMS databases: SWGDRUG Mass Spectral Library [6] and Cayman Chemical Spectral Library [14]. Mass spectra obtained from the SWGDRUG database (Versions 3.11) were used as the training set while mass spectra from Cayman Spectral Library (Cayman Chemical Company (CCC) Ann Arbor, MI, USA) were used as a test set. Mass spectral data were cropped to include the *m*/*z* range of 40 to 300 before being used for model development in this study. First, the data space of the training set was evaluated using PCA and HCA before the development of binary classification models. The primary goal of PCA was to see if any trends between the cannabinoids and other drugs were evident in mass spectral data. The PCA score plot (Figure 2) shows some separation between these two classes suggesting that supervised learning methods such as PLS-DA can be employed for discrimination. Although some samples seem to be outliers, these samples were not removed at this stage due to them having possibly important spectral information that can be useful in PLS-DA analysis. The HCA was used to investigate the possible grouping of cannabinoids. The dendrogram generated using the Wards method shows the clustering of classical cannabinoids. Additional clustering was observed for functional groups including the naphthyl head group, indole, and indazole/azaindole core groups (see Figure A2). These results show the feasibility of using supervised learning methods such as PLS-DA for building classification models.

### 2.2. Partial Least Squares Discriminant Analysis

#### 2.2.1. General Overview of the Binary Classification System

**Figure 3:**
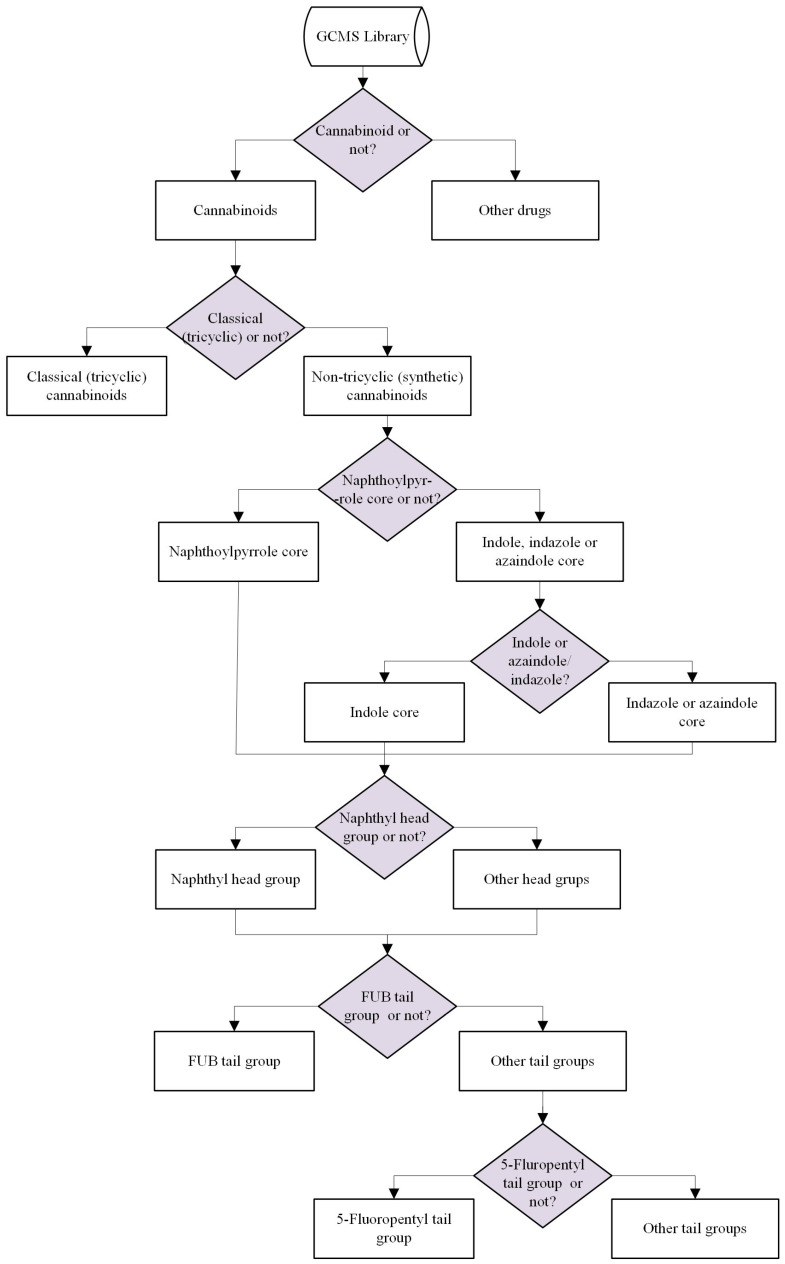
General overview of the binary classification system.

The aim of this study is to develop a classification system capable of identifying previously unknown cannabinoids and providing potential structural information about these compounds. The flow chart shown in Figure 3 illustrates the application of this system. In summary, the classification system begins by distinguishing cannabinoids from other drugs. Then, the classical cannabinoids that contain the tricyclic core structures are separated from synthetic cannabinoids that do not contain tricyclic core structures. Synthetic cannabinoids are further divided into those with a naphthoylpyrrole core and those with indole, indazole, or azaindole core groups. In the next step, compounds containing indole core groups are separated from compounds that have indazole or azaindole core groups. Then, naphthyl head groups containing compounds are separated from those with other head groups. Finally, two models were developed for FUB and 5-Fluoropentyl tail groups. For a suspected drug molecule with an unknown identity, the GCMS data should be projected onto the binary models to obtain structural information.

#### 2.2.2. Cannabinoids versus Other Drugs

**Figure 4:**
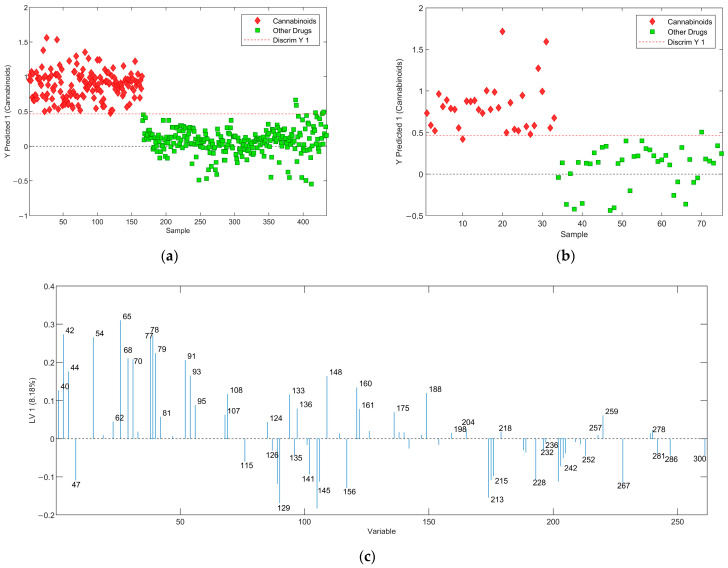
The Results of PLS-DA analysis of cannabinoids and other drugs using GA as a feature selection method: (**a**) PLS-DA score plot of the training set; (**b**) PLS-DA score plot of the prediction set; (**c**) LV1 with the weights. Here, the x-axis is the *m*/*z* ratio.

The first classification model was developed to discriminate cannabinoids from other drugs using PLS-DA. A genetic algorithm (GA) was employed for feature selection to identify the most relevant features (*m*/*z* values) for distinguishing between classes. Given that the mass spectral data in this study range from *m*/*z* 30 to 400, many features may not possess any discriminatory value, while some could introduce noise into the classification process [15]. The decision threshold is denoted by a red dashed line in the PLS-DA score plot, and samples with discrimination scores above the decision threshold are classified as cannabinoids, while those with scores below the threshold are classified as other drugs. LV1 of this model (Figure 4c) shows the use of a limited number of variables that can be associated with the use of GA for feature selection. Matthew’s correlation coefficient (MCC) was primarily used to determine the predictive ability of the developed models as this metric is reported as the most reliable evaluator for binary classifications. Additionally, the true positive rate (TPR), true negative rate (TNR), and F1 score were calculated for all the models in this study. For the model developed for cannabinoids versus other drugs, all the above matrices were found to be greater than 0.9700, demonstrating a very high predictive ability (see Table 1).

In a recent review, Feeny et al. summarized the common mass fragments of different drug classes [1]. This information along with other published research is used frequently in this article to explain the presence of certain mass fragments in LVs. Three LVs were used to develop PLS-DA plots shown in Figure 4, and the analysis of LV1 shows that it correlates with overall class separation, while most variables are important in distinguishing fentanyl derivatives from other drugs. Additionally, sample scores suggest that the second LV (LV2) is important in discriminating cathinones and amphetamines from cannabinoids, while LV3 plays an important role in separating non-fentanyl-related opioids (morphine-related) from cannabinoids. The correlation between the drug classes and the variables (*m*/*z*) with the highest weight in LV1 is explained below. Ions at *m*/*z* of 42, 77, 91, 95, 160, and 259 are reported as frequently observed fragments in the fentanyl class drugs while the peaks at *m*/*z* of 44, 65, 77, 91, and 95 are reported as common fragments of cathinone-related compounds [1,16]. Peaks at *m*/*z* of 42, 44, 70, 77, and 91 can be related to non-fentanyl opioids. Additionally, peaks at *m*/*z* of 44, 77, 91, and 259 are reported as fragments of phenethylamines, and peaks at *m*/*z* of 44, 77, 160, and 161 are fragments reported for tryptamine-related drugs [1]. These peaks are positively associated with the X loadings in LVs while this loading vector is negatively associated with the Y loadings for cannabinoid-related compounds. This means that when a particular compound has strong peaks at *m*/*z* values that are positively associated with *m*/*z* values on an LV, that compound will likely be classified as an “other drug”, whereas if a compound has strong peaks at *m*/*z* values that are negatively associated with an LV, that compound will most likely be classified as a cannabinoid. For example, the peaks shown in LV1 on the negative side (bottom) correlate with the cannabinoid drug class. Fragment peaks are seen at *m*/*z* of 144 and 145 and are common fragments of most synthetic cannabinoids that contain indole and indazole core groups, respectively. The fragments of *m*/*z* of 214 and 215 correlate to the non-fluorinated core structure while the peak at 232 correlates to the fluorinated core structure of synthetic cannabinoids that are formed by the cleavage between the carbonyl and benzyl carbon atoms. Additionally, the peaks around *m*/*z* of 232 are common fragments of classical cannabinoids that contain tricyclic structures such as THC [1]. The interpretation of other major peaks shown in the LVs was not conducted as they can be related to other drug classes included in this study. Matthew’s correlation coefficient (MCC) was primarily used to determine the predictive ability of the models as this metric is reported as the best evaluator for binary models. Table 1 shows MCC and other commonly used matrices calculated for calibration, cross-validation, and external validation. For all these matrices, the values closer to one show better predictive ability. Five false positives were observed in the calibration model including buprenorphine (opioid), butorphanol (opioid), 2-C-B-fly (phenethylamine), 4-AcO-DIPT (tryptamine), and 5-MeO-DiPT (tryptamine). Although buprenorphine is structurally similar to morphine-related compounds, the mass spectral comparison shows significant differences between morphine-related compounds and buprenorphine. Additionally, butorphanol, a morphinan, shows a mass spectrum with a very strong peak at *m*/*z* of 272 with many smaller peaks scattered throughout the spectral range of interest. Although misclassification cannot necessarily be correlated with *m*/*z* values, the differences between the spectra of these compounds compared to the drug classes included in this classification problem could contribute to the misclassification. The mass spectrum of the false-positive sample 2-C-B-fly contains peaks at *m*/*z* of 115 and 145 that are commonly present in cannabinoids. This explains the misclassification of this sample. The misclassification of 4-AcO-DIPT and 5-MeO-DiPT was not able to be explained using the LVs. There were two misclassifications in the external validation including ADB-BICA (false negative) and βOH-2C-B (false positive). The mass spectrum of ADB-BICA has a base peak at *m*/*z* of 91 that can be related to the fragment forms by the cleavage of the 1-benzyl tail group of this compound. According to the LV1 (Figure 4c), the peak at 91 is associated with non-cannabinoid drugs. Interestingly, a correctly predicted test set sample, ADB-BINACA, contains the same tail group; thus, the peak at *m*/*z* of 91. However, the mass spectrum of this sample shows a peak at *m*/*z* of 145, a common fragment forms from the indole core containing cannabinoids and consequently predicts ADB-BINACA as a cannabinoid. The misclassification of βOH-2C-B can be associated with the presence of peaks at *m*/*z* of 215 and 232 that coincide with synthetic and classical cannabinoid structures respectively.

#### 2.2.3. Classical Versus Synthetic Cannabinoids

**Figure 5:**
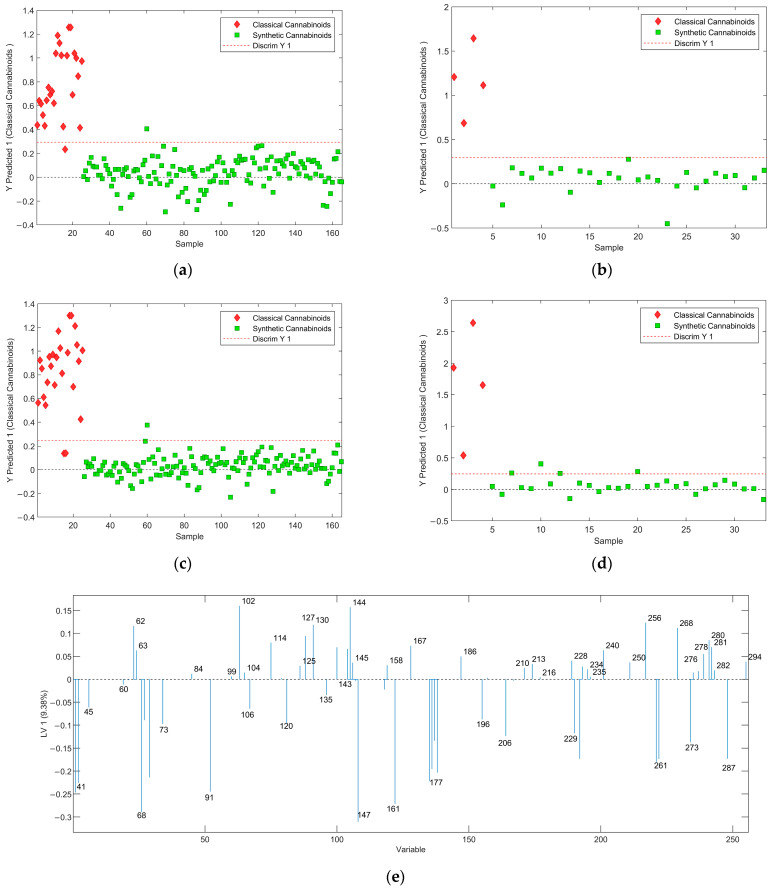
The Results of PLS-DA analysis of synthetic cannabinoids and classical cannabinoids: (**a**) score plot of the training set and (**b**) prediction set without using variable selection methods; (**c**) score plot of the training set and (**d**) prediction set with using GA as the variable selection method. (**e**) LV1 of the weights for the model developed using GA as the variable selection method.

Cannabinoids can be divided into three major classes including classical cannabinoids (or phytocannabinoids), endocannabinoids, and synthetic cannabinoids. For this work, only classical cannabinoids and synthetic cannabinoids were selected due to their abundance in forensic casework. Here a PLS-DA classifier was developed to separate synthetic cannabinoids from classical cannabinoids. It should be noted that the classical cannabinoid class includes some synthetic compounds such as HU-210 and HU-211 that share the tricyclic structure of classical cannabinoids [17]. PLS-DA was performed with and without using GA as the variable selection method using two LVs. High accuracy is observed when GA was not used for feature selection (see Figure 5) and the predictive ability of the model decreased when GA was used. This can be attributed to the overfitting in feature selection. Classical cannabinoid mass spectra usually contain a very large number of peaks compared to other drug molecules included in the study [1]. Although GA selects important variables for this classification, it may not have selected some vital variables that are present in the prediction samples causing them to misclassify. For the PLS-DA developed without using GA, the MCC was found to be 0.6570 for the prediction set, whereas MCC was 1.0000 (maximum possible predictability) for the models developed without using GA.

The analysis of LV1 of the PLS-DA after feature selection reveals that there are some important *m*/*z* contributed to the discrimination between classical and synthetic cannabinoids (see Figure 5e). Fragments with *m*/*z* of 41, 69, 73, 91, 175, 231, and 260 have been reported as common fragments for classical cannabinoids [1,18]. Furthermore, the peaks at *m*/*z* of 144 and 145 are common fragments of synthetic cannabinoids with indole and indazole/azaindole core groups, respectively. The peak at *m*/*z* of 127 can be correlated with the fragmentation of the naphthyl group, which is a common functional group in the JWH series of synthetic cannabinoids [1,19,20]. The peak at *m*/*z* of 186 can be related to an n-pentylindole fragment, which is a product of synthetic cannabinoids that contain both indole core and pentyl tail group [21].

#### 2.2.4. Core Group Analysis—Naphthoylpyrrole Versus Indole, Indazole, and Azaindole

There are four main core groups present in synthetic cannabinoids: indole, indazole, azaindole, and naphthoylpyrrole. A few other core groups are also reported in synthetic cannabinoids, but the four core groups mentioned above were selected as they were the most abundant, and the mass spectral database contained a sufficient number of compounds in each class to develop classification models. The naphthoylpyrrole core group has a pyrrole ring that is usually connected to a naphthyl group while the other three groups have a double ring system. Due to these differences, it was logical to separate naphthoylpyrrole-containing compounds from other cannabinoids that contain indole, indazole, and azaindole core structures. Here, the PLS-DA was performed using three LVs. The analysis of LV1 (Figure S1c) of the GA-PLS-DA shows a little correlation between the selected features (*m*/*z*) and the structure differences between the classes. However, the peak at *m*/*z* 145 can be related to the indole core group. Both models developed with and without GA show 100% accuracy in predicting the test set (Table 1).

#### 2.2.5. Cannabinoids Containing Indole Core Group versus Indazole/Azaindole Core Groups

**Figure A3:**
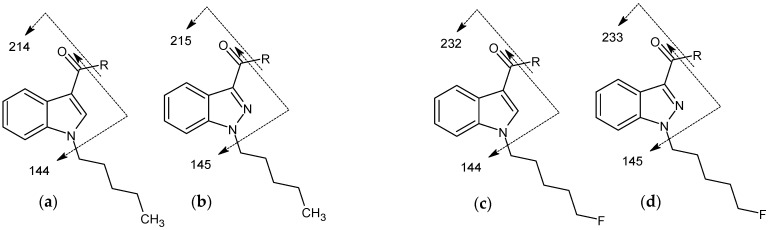
Formation of fragments of interest for indole and indazole core groups: (**a**) Indole core group with pentyl tail group. (**b**) Indazole core group with pentyl tail group. (**c**) Indole core group with 5-fluoropentyl tail group. (**d**) Indazole core group with 5-fluoropentyl tail group.

Indole- and indazole-containing drugs are common in synthetic cannabinoids. These both contain bicyclic structures consisting of a benzene ring combined with pyrrole or pyrazole rings (see Figure A3). Due to the second nitrogen atom present in the pyrazole moiety, indazole-containing compounds form one *m*/*z* heavier fragments compared to that of indole-containing compounds. Additionally, azaindole is also present in synthetic cannabinoid structures. It is reported that indazole- and azaindole-containing cannabinoids undergo similar fragmentation patterns during the ionization producing fragments with similar *m*/*z* ratios [16]. Therefore, cannabinoids containing azaindole and indazole core groups were combined. Discrimination of indoles from indazoles/azaindoles was straightforward and the PLS-DA plots were generated using 2 LVs. The positive peaks of LV1 (top) are associated with indoles and the negative peaks (bottom) are associated with indazoles and azaindoles according to the Y loading vectors calculated by the PLS-DA model (see Figure S2c. The important *m*/*z* values include 144/155, 214/215, 232/233, 242/243, and 256/257. The one *m*/*z* difference observed is due to the second nitrogen atom present in indazole/azaindole functional groups. Peaks at 144/155 and 214/215 can be associated with the indole and indazole/azaindole core with a pentyl tail. Additionally, the peaks at 232/233 can be associated with indole/indazole core groups attached to a 5-fluoropentyl tail group. Figure A2 shows the formation of fragments corresponding to *m*/*z* of 144/145, 214/215, and 232/233 [20,21,22,23]. The calibration model shows no misclassifications, while two external validation samples (CUMYL-PIPETINACA and ADB-BINACA) that contain an indazole core group were misclassified as indole-containing compounds. CUMYL-PIPETINACA has a piperidine-containing tail group. The mass spectrum of this compound shows the base peak at *m*/*z* of 98 due to the cleavage of the piperidine-containing tail group and interestingly all other peaks show very little abundance. Furthermore, ADB-BINACA has a methyl benzene tail group, thus showing an intense peak at *m*/*z* of 91. Additionally, the peak at *m*/*z* of 145 that corresponds to the indazole core is relatively small and the peak at *m*/*z* of 235 can be correlated with the combined mass of the linking group, core group, and tail group. No major peaks that are present in the mass spectra of either compound can be related to the classes based on LVs. Although this information cannot be used to justify the misclassifications, it shows the limitations of this approach.

#### 2.2.6. Cannabinoids Containing Naphthyl Head Group

**Figure A4:**
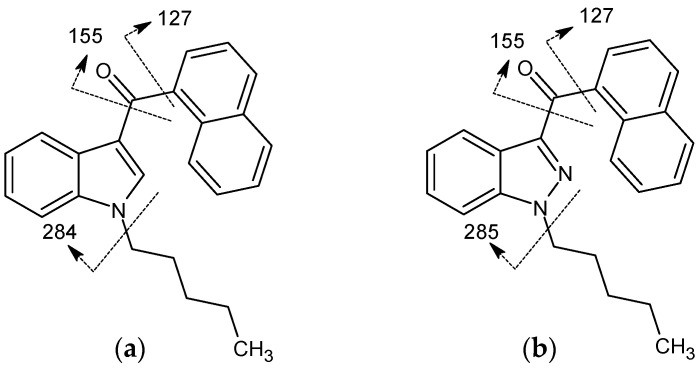
Formation of fragments of interest for cannabinoids containing naphthyl head group: (**a**) with indole, (**b**) with indazole core groups.

Cannabinoids containing the naphthyl functional group (naphthoylindoles) are one of the earliest groups of synthetic cannabinoids that were synthesized, and they are predominantly present in the JWH series of designer cannabinoids [12]. Two LVs were used in PLS-DA model development and the analysis of LV1 (Figure S3c) reveals the presence of important peaks for discrimination of classes. The positive peaks of LV1 (top) are associated with other head groups, while the negative peaks (bottom) are associated with naphthyl-containing cannabinoids according to the Y loading vectors calculated by the PLS-DA model. The peak at *m*/*z* of 127 correlates with the cleavage of the naphthyl group from the linking group, while the peak at *m*/*z* of 155 correlates with the cleavage of the naphthyl group and the carbonyl linking group from the core group. The peaks at *m*/*z* of 284 and 285 can be correlated with the cleavage of the tail group of most naphthyl head groups containing cannabinoids with indole and indazole core groups, respectively [16,19,24] (see Figure A4). Four misclassifications were observed including one false positive (pravadoline) and three false negatives (MN-18, 5F-NNEI 2′-naphthyl isomer, and AM1220). Pravadoline has a 2-methyl group on the indole core group. This is not a common functional group found in synthetic cannabinoids. The mass spectrum of this compound shows a very strong peak at *m*/*z* of 100 with no other major peaks explaining the misclassification. Both MN-18 and 5F-NNEI 2′-naphthyl isomers have a carboxamide linking group while most naphthyl-containing compounds (JWH series) have the methanone linker. Therefore, the misclassification of these compounds can be associated with the presence of an uncommon linker group attached to the naphthyl head group. The mass spectrum of AM1220 shows a very strong peak at *m*/*z* of 98 with no other significant peaks causing misclassification.

#### 2.2.7. Cannabinoids Containing 4-Fluorobenzyl (FUB) Tail Group

**Figure A5:**
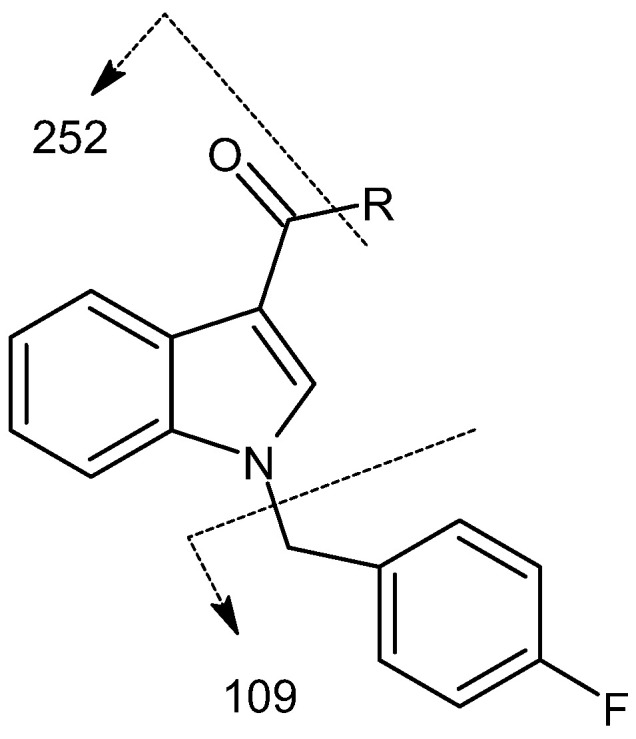
Formation of fragments of interest for cannabinoids containing FUB tail group.

The PLS-DA classification model developed using GA showed 100% accuracy. This model was developed by using one LV, and it shows a selection of the most discriminatory *m*/*z* ratios including *m*/*z* of 109 and 252 (Figure S4). The peak at *m*/*z* of 109 is the base peak of the majority of compounds in this class and this peak correlates with the cleavage of the FUB group from the core group. The peak at *m*/*z* of 252 can be associated with the cleavage of the indole core and the FUB functional group from the linking group (see Figure A5) [22,25]. No misclassifications in calibration or prediction were observed.

#### 2.2.8. Cannabinoids Containing 5-Fluoropentyl (5F-Pentyl) Tail Group

**Figure A6:**
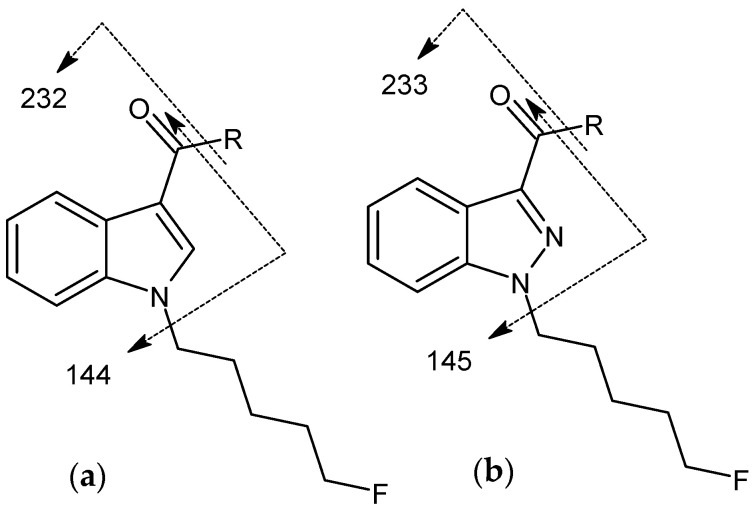
Formation of fragments of interest for cannabinoids containing 5-fluoropentyl tail group: (**a**) with indole, (**b**) with indazole core groups.

The 5F-pentyl tail group is a commonly found tail group in synthetic cannabinoids [9]. These molecules tend to lose the head group during ionization and the indole-containing fragments will have an *m*/*z* of 232. Whereas the indazole- or azaindole-containing compounds will have an *m*/*z* of 233 for the same fragment [19,22] (see Figure A6). Two false negatives were observed in the calibration model including AM1235 and THJ 2201 (Figure S5). These compounds are structurally similar to JWH-type cannabinoids by having a naphthyl head group. As explained in Section 2.2.6, these compounds show intense peaks at *m*/*z* of 127 and 155 after the cleavage of the head group with or without the carbonyl linker. Additionally, these compounds tend to lose the tail group as shown in Figure A6 causing the absence of peaks at *m*/*z* of 232 or 233. Furthermore, AM1235 contains a nitro group attached to the indole core, which can explain the absence of the peak at *m*/*z* of 232 that causes misclassification. Three false positives were observed in the calibration including 4-fluoro AMB, AB PINACA N (3-fluoropentyl isomer), and MBDB-CHM7AICA (Figure S5). Both 4-fluoro AMB and AB PINACA N (3-fluoropentyl isomer) contain a 4-fluoropentyl tail group that shares the same mass as the 5-fluoropentyl tail group. Therefore, these compounds have base peaks at *m*/*z* of 232 making them predicted as 5F-pentyl containing cannabinoids. The misclassification of MBDB-CHM7AICA can be explained using the peak observed at *m*/*z* of 233 in the mass spectrum of this compound.

## 3. Materials and Methods

### 3.1. Mass Spectral Data

All mass spectra were obtained from freely available databases. Mass spectra obtained from the SWGDRUG database (Versions 3.11) [6] were used as the training set data while mass spectra from Cayman Spectral Library (Cayman Chemical Company (CCC), Ann Arbor, MI, USA) [14] were used as test set data. Here, the compounds included in the test set were not included in the training set. The training set included 165 cannabinoids and 269 other drugs including, 173 fentanyl derivatives, 27 opioids, 48 cathinones, 7 tryptamines, and 14 phenylethylamines. The test set from CCC included 45 cannabinoids and 32 other drugs. The number of compounds included in each model can be found in Table A1 and Table A2. All mass spectra included in this study were collected using electron ionization (EI) mass spectroscopy and more information on the collection method can be found elsewhere [1,6,14].

### 3.2. Software

All models were developed using PLS_Toolbox 8.7.1 (Eigenvector Research Inc., Manson, WA, USA), which performs calculations on MATLAB (MATLAB 2024a, by Mathworks Inc., Natick, MA, USA) programming language.

### 3.3. Data Processing and Analysis

Mass spectra were cropped to retain only data from *m*/*z* of 40 to 300. The data obtained from CCC was scaled to match the relative abundance of that of the SWGDRUG library. All data were autoscaled before analysis. Principal Component Analysis (PCA), and partial least square discriminant analysis (PLS-DA) were performed on autoscaled data. Cross-validation was performed for each model using Venetian blinds with 10 data splits. Genetic algorithm (GA) was used as a variable selection method. The GA parameters include a population size of 64, a window width of 1, and a mutation rate of 0.005. All the GA runs were carefully monitored to select the optimum number of generations so as not to overfit the models. It was found that most models needed from 20 to 50 generations, and they tend to overfit and decrease the accuracy of prediction results after 50 generations.

### 3.4. Evaluation of Classification Models

All models were evaluated using calibration, cross-validation, and external validation methods. The classification matrices including accuracy, sensitivity (TPR), specificity (TNR), F1 score, and Matthew’s correlation coefficient were calculated as follows [26,27].

- Accuracy—proportion of cases correctly allocated,

(1) $$Accuracy=\frac{\left(TP+TN\right)}{\left(TP+TN+FN+FP\right)}$$

- Sensitivity (TPR)—proportion of positive cases that were correctly identified,

(2) $$TPR=\frac{TP}{\left(TP+FN\right)}$$

- Specificity (TNR)—proportion of negative cases that were classified correctly

(3) $$TNR=\frac{TN}{\left(TN+FP\right)}$$

- F-1 score—can be interpreted as a harmonic mean of the precision and sensitivity.

(4) $$F1\;Score=\frac{2TP}{2TP+TP+FP+FN}$$

- Matthew’s Correlation Coefficient (MCC)—measures the correlation between real and predicted values and reported as the best evaluator for binary classification models [21].

(5) $$MCC=\frac{\left(TP*TN)-(FP*FN\right)}{\sqrt{\left(TP+FP\right)*\left(TP+FN\right)*\left(TN+FP\right)*(TN+FN)}}$$

where

TP = true positives—actual positives that are correctly predicted positives;

FN = false negatives—actual positives that are wrongly predicted negatives;

TN = true negatives—actual negatives that are correctly predicted negatives;

FP = false positives—actual negatives that are wrongly predicted positives.

## 4. Conclusions

This research has shown the ability to use mass spectral data of known compounds to aid in identifying newly synthesized previously unknown cannabinoids. Classification models developed using readily available mass spectral data showed successful discrimination of cannabinoid-related compounds from other drug classes. Then, a classification model was developed to discriminate classical (THC-related) from synthetic cannabinoids with high accuracy. Further models were developed based on the most abundant functional groups. These groups include core groups such as indole, indazole, azaindole, naphthoylpyrrole, naphthyl head group, and tail groups including 4-fluorobenzyl (FUB) and 5-Fluoropentyl (5-F). Cross-validation was used to validate the developed models, and all developed models showed very high accuracy. The prediction set used to further evaluate the robustness of these models consists of a separate sample cohort that was not included in the training set and the accuracy, F1 score and MCC shows the applicability of this system. Furthermore, the analysis of LVs and loadings illustrates the basis of discrimination of each model. The information provided by the LVs and loadings can be used to find the unique and most abundant mass-to-charge ratios (*m*/*z*) of each drug class. The analysis of misclassifications shows the limitations of this approach. Since the LVs use *m*/*z* values that are unique to a given class of molecules, the minor structural changes that alter the fragmentation pattern of a molecule can affect the predictive ability. The variables used in these models are mass-to-charge ratios, and they are present as discrete values when low-resolution mass spectrometers are used. During the ionization process, two different drug molecules that belong to two different classes can generate fragments with similar masses. If these masses are important in discriminating those two drug classes, these samples can be misclassified. Although the methods described in this article would not eliminate the use of structural determination techniques such as NMR and HRMS, the detection of novel drugs in a variety of matrices and the identification of functional groups present on these molecules using such methods would greatly benefit the forensic analysis process.

## Figures and Tables

**Table 1:** Figures of merit obtained for the calibration and prediction data for models developed using GA-PLS-DA.

Model	Accuracy	TPR *	TNR **	F1 Score	MCC ***
Cannabinoids vs. other drugs					
Calibration	0.9885	1.0000	0.9814	0.9851	0.9760
Cross Validation	0.9816	0.9939	0.9740	0.9762	0.9620
External validation	0.9733	0.9697	0.9762	0.9697	0.9460
Classical vs. synthetic cannabinoids					
Calibration	0.9886	0.9200	0.9929	0.9388	0.9280
Cross-validation	0.9697	0.9200	0.9786	0.9020	0.8840
External validation	0.8788	1.0000	0.8620	0.6667	0.6570
Naphthoylpyrrole vs. other core groups					
Calibration	1.0000	1.0000	1.0000	1.0000	1.0000
Cross-validation	0.9690	1.0000	0.9457	0.9843	0.8430
External validation	1.0000	1.0000	1.0000	1.0000	1.0000
Azaindole/Indazole vs. Indole					
Calibration	1.0000	1.0000	1.0000	1.0000	1.0000
Cross-validation	0.9845	1.0000	0.9753	0.9796	0.9680
External validation	0.9253	0.8750	1.0000	0.9333	0.8600
Naphthyl vs. other head groups					
Calibration	0.9714	0.9318	0.9896	0.9535	0.9330
Cross-validation	0.9714	0.9318	0.9896	0.9535	0.9330
External validation	1.0000	1.0000	1.0000	1.0000	1.0000
FUB vs. other tail groups					
Calibration	1.0000	1.0000	1.0000	1.0000	1.0000
Cross-validation	1.0000	1.0000	1.0000	1.0000	1.0000
External validation	1.0000	1.0000	1.0000	1.0000	1.0000
5-Fluoropentyl vs. other tail groups					
Calibration	0.9643	0.9394	0.9720	0.9254	0.9020
Cross-validation	0.9571	0.9394	0.9626	0.9118	0.8840
External validation	1.0000	1.0000	1.0000	1.0000	1.0000

* TPR = True positive rate, ** TNR = True negative rate, and *** MCC = Matthew’s correlation coefficient.

## Data Availability

All data presented are available at request from the corresponding author.

## Supplementary Materials

The following supporting information can be downloaded at: https://www.mdpi.com/article/10.3390/molecules29194646/s1, Figure S1. The Results of PLS-DA analysis of cannabinoids with naphthoypyrrole core and cannabinoids with indole, azaindole, or indazole core groups. Figure S2. The Results of PLS-DA analysis of indole core containing cannabinoids versus azaindole or indazole core containing cannabinoids, Figure S3. The Results of PLS-DA analysis of cannabinoids containing naphthyl head group versus cannabinoids containing other head groups, Figure S4. The Results of PLS-DA analysis of cannabinoids containing FUB tail group versus cannabinoids containing other head groups, and Figure S5. The Results of PLS-DA analysis of cannabinoids containing 5-Fluoro tail group versus cannabinoids containing other tail groups.

## Appendix A

**Table A1:** All samples were used in model development.

Drug Type	SWGDRUG Database	Cayman Chemical Company Database
	(Training Set)	(Prediction Set)
Cannabinoids	165	33
Fentanyl	173	11
Opioids	27	8
Cathinones	48	10
Tryptamines	7	8
Phenylethylamines	14	5
Total	434	75

**Table A2:** Calibration and external validation samples used in model development.

Model	Number of LVs Used	Class	Training Set	Prediction Set
Cannabinoids vs. other drugs	3	Cannabinoids	165	33
		Other drugs	269	42
Classical vs. synthetic cannabinoids	2	Classical cannabinoids	25	4
		Synthetic cannabinoids	140	29
Naphthoylpyrrole vs. other core groups	3	Naphthoylpyrrole	11	2
		Other core groups	129	27
Azaindole/Indazole vs. Indole	2	Indole core	48	16
		Azaindole/Indazole core	81	11
Naphthyl vs. other head groups	2	Naphthyl head group	44	2
		Other head groups	96	27
FUB vs. other tail groups	1	FUB tail group	9	9
		Other tail groups	131	20
5-Fluoropentyl vs. other tail groups	3	5-Fluoropentyl tail group	33	1
		Other tail groups	107	28
