# Supplementary material for: Classification of Mass Spectral Data to Assist in the Identification of Novel Synthetic Cannabinoids
Source: Molecules. 2024 Sep 30;29(19):4646. doi: 10.3390/molecules29194646 (PMC11478242; doi:10.3390/molecules29194646)
Supplement: Supplementary file 1 [file molecules-29-04646-s001.zip › molecules-3192806-supplementary.pdf]

Classification of mass spectral data to assist in the identification of novel synthetic cannabinoids.

Kristopher C. Evans-Newman, Garion L. Schneider and Nuwan T. Perera

Supplementary Materials

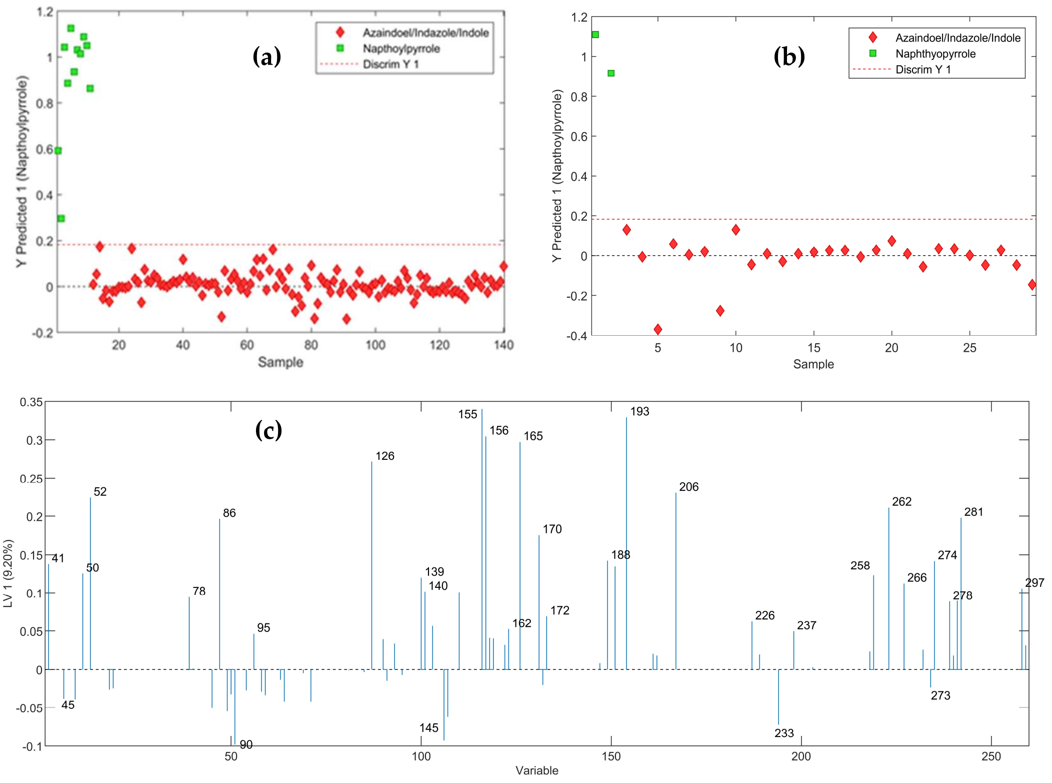

Figure S1. The Results of PLS-DA analysis of cannabinoids with naphthoypyrrole core and cannabinoids with indole, azaindole, or indazole core groups: (a) score plot of the training set and (b) prediction set using GA as the variable selection method. (c) LV1 with the weights.

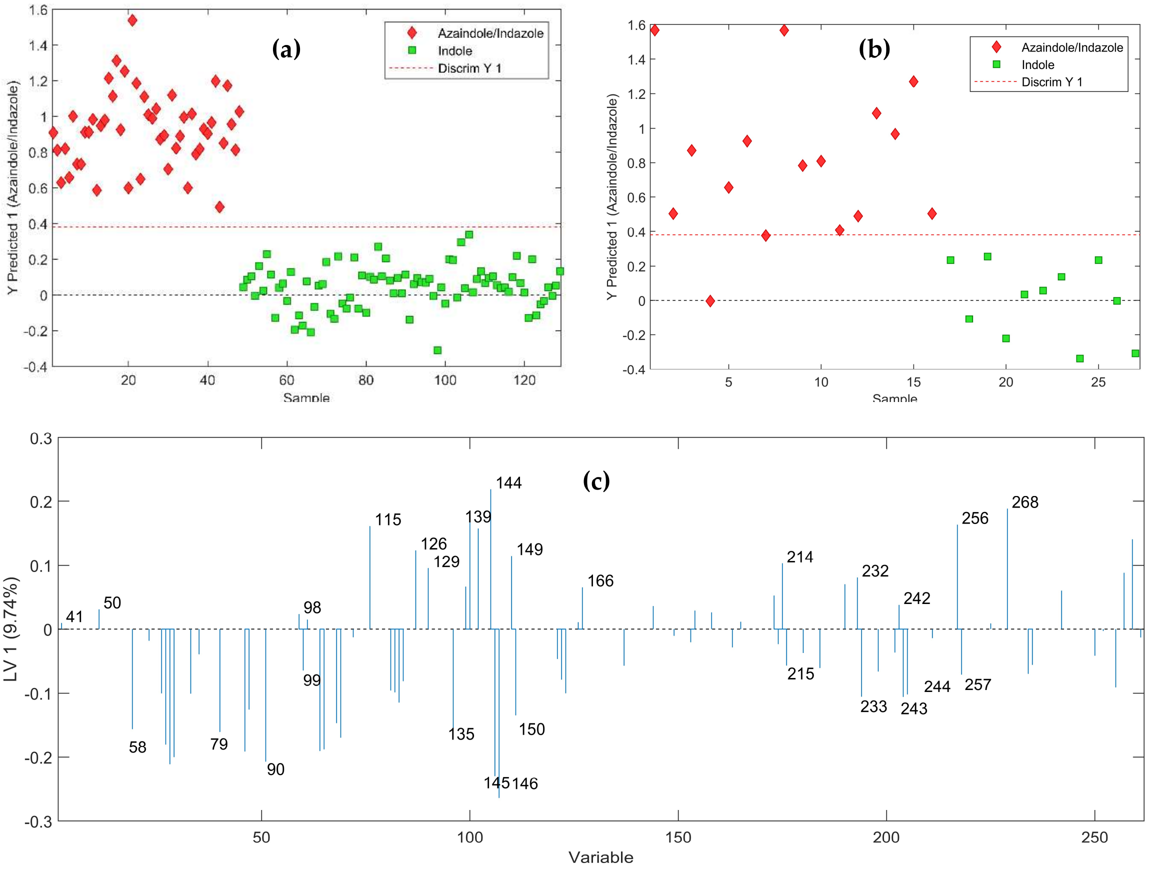

Figure S2. The Results of PLS-DA analysis of indole core containing cannabinoids versus azaindole or indazole core containing cannabinoids: (a) score plot of the training set and (b) prediction set using GA as the variable selection method. (c) LV1 with the weights.

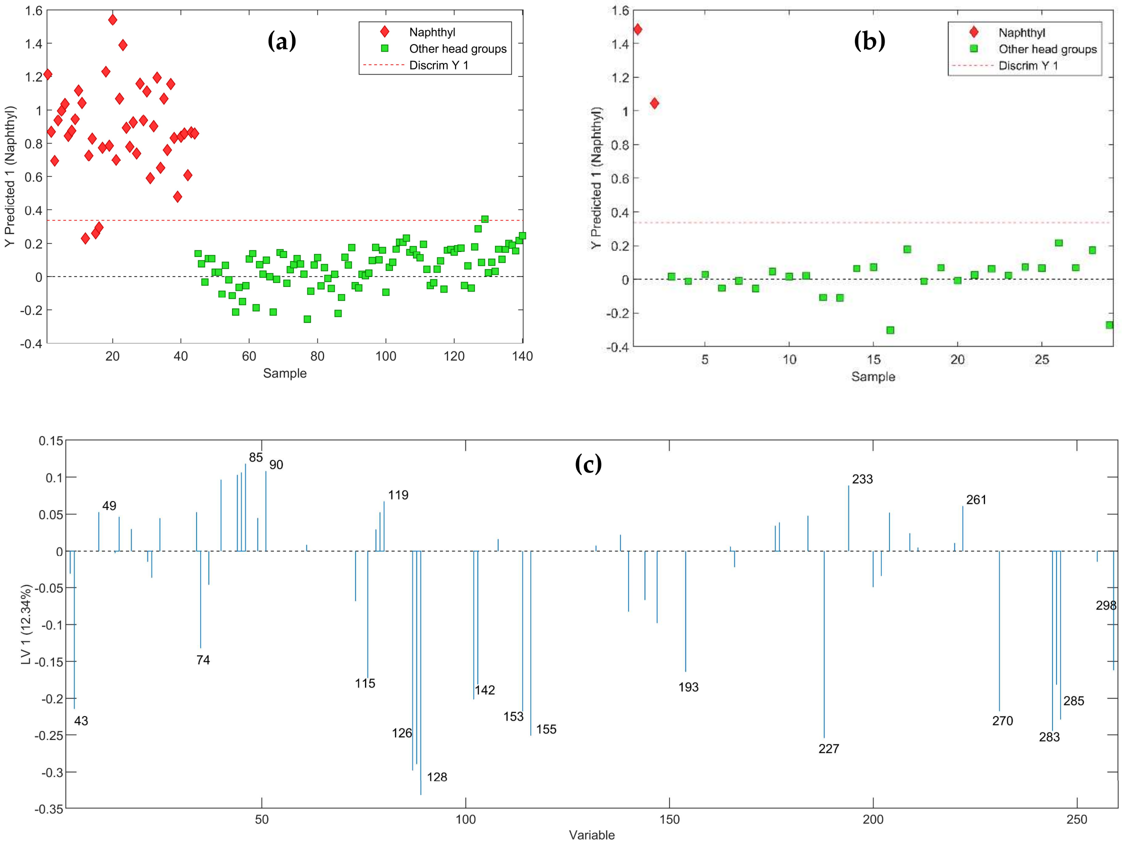

Figure S3. The Results of PLS-DA analysis of cannabinoids containing naphthyl head group versus cannabinoids containing other head groups: (a) score plot of the training set and (b) prediction set using GA as the variable selection method. (c) LV1 with the weights.

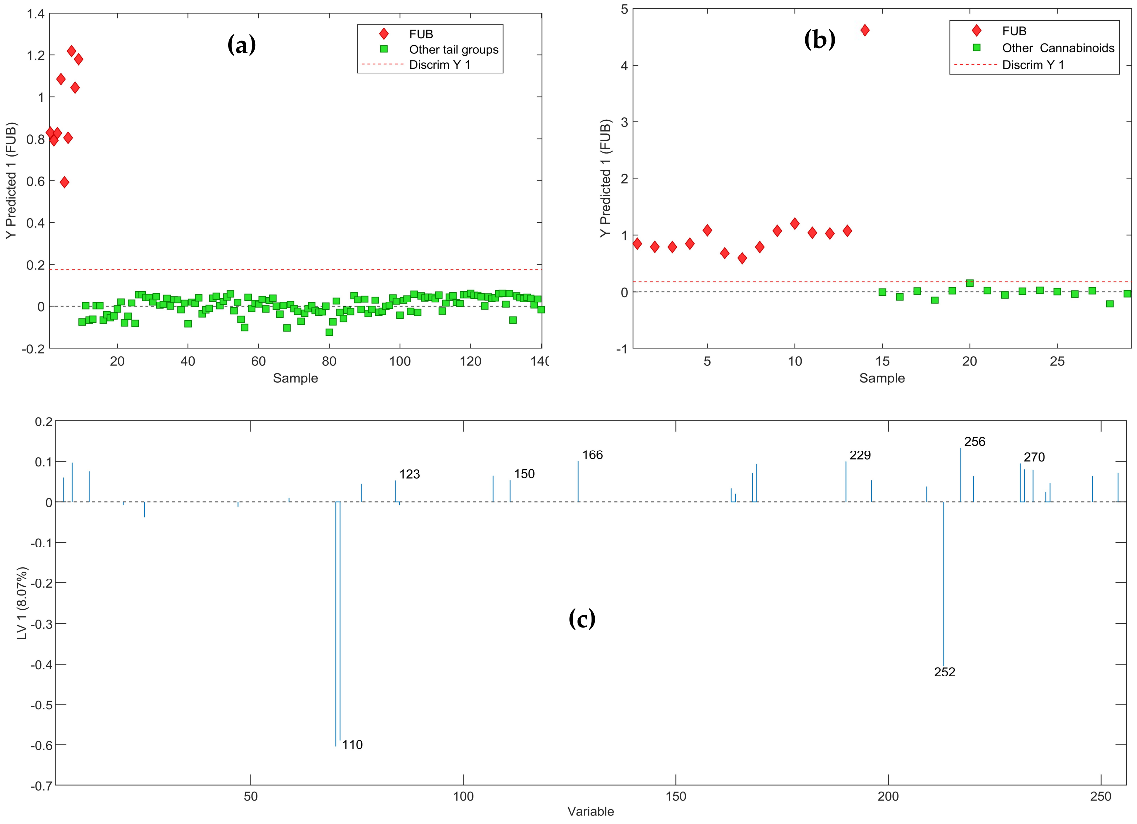

Figure S4. The Results of PLS-DA analysis of cannabinoids containing FUB tail group versus cannabinoids containing other head groups: (a) score plot of the training set and (b) prediction set with using GA as the variable selection method. (c) LV1 with the weights.

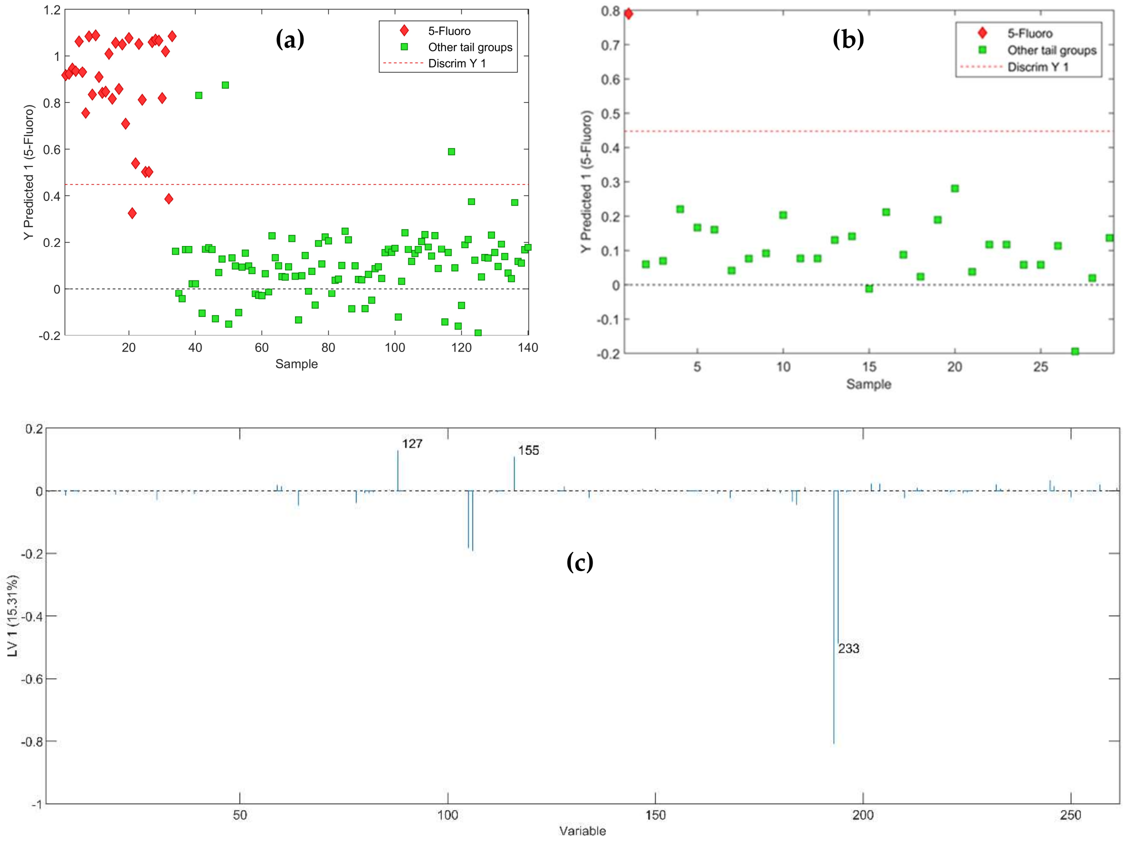

Figure S5. The Results of PLS-DA analysis of cannabinoids containing 5-Fluoro tail group versus cannabinoids containing other tail groups: (a) score plot of the training set and (b) prediction set using GA as the variable selection method. (c) LV1 with the weights.
